# Magnetoconductivity in quasiperiodic graphene superlattices

**DOI:** 10.1038/s41598-020-78479-9

**Published:** 2020-12-04

**Authors:** M. de Dios-Leyva, A. L. Morales, C. A. Duque

**Affiliations:** 1 Department of Theoretical Physics, University of Havana, San Lázaro y L, Vedado, 10400 Havana, Cuba; 2 Grupo de Materia Condensada-UdeA, Instituto de Física, Facultad de Ciencias Exactas y Naturales, Universidad de Antioquia UdeA, Calle 70 No. 52-21, Medellín, Colombia

**Keywords:** Physics, Condensed-matter physics, Quantum Hall, Semiconductors, Surfaces, interfaces and thin films

## Abstract

The magnetoconductivity in Fibonacci graphene superlattices is investigated in a perpendicular magnetic field *B*. It was shown that the *B*-dependence of the diffusive conductivity exhibits a complicated oscillatory behavior whose characteristics cannot be associated with Weiss oscillations, but rather with Shubnikov-de Haas ones. The absense of Weiss oscillations is attributed to the existence of two incommensurate periods in Fibonacci superlattices. It was also found that the quasiperiodicity of the structure leads to a renormalization of the Fermi velocity $v_{F}$ of graphene. Our calculations revealed that, for weak *B*, the dc Hall conductivity $\sigma _{yx}$ exhibits well defined and robust plateaux, where it takes the unexpected values $\pm 4e^{2}/\hslash \left( 2N+1\right)$, indicating that the half-integer quantum Hall effect does not occur in the considered structure. It was finally shown that $\sigma _{yx}$ displays self-similarity for magnetic fields related by $\tau ^{2}$ and $\tau ^{4}$, where $\tau$ is the golden mean.

## Introduction

After the experimental realization of graphene^1^, a great deal of attention has been paid to the study of its magnetotransport properties. The early studies^2–5^ revealed that, when monolayer graphene is subject to a perpendicular magnetic field $\overrightarrow{B}$, the magnetoconductivity $\sigma _{\alpha \beta }$ exhibit unusual properties, such as the anomalous integer quantum Hall effect. The latter is directly associated with the presence of the $n=0$ Landau level (LL) in the nonequidistant and highly degenerate energy spectrum, $E_{n}=sgn(n)\hslash \omega _{C}\sqrt{\left| n\right| }$, of single layer graphene^6^, where $\hslash \omega _{C}=\sqrt{2}\hslash v_{F}/l_{B}$ is the cyclotron energy, $l_{B}=\sqrt{\hslash c/eB}$ the magnetic length, $v_{F}$ the Fermi velocity and $n=0,\pm 1,\pm 2,\ldots$ the energy quantum number.

In subsequent studies, the effects of a one-dimensional (1D) periodic potential $V_{P}(x)$ on the LLs and on the corresponding magnetoconductivity characteristics were investigated. In this case, the degeneracy of each LL is in general lifted, which leads to the formation of magnetic subbands. Further, it should be remarked that the effects of $V_{P}(x)$ on the magnetoconductivity are usually described in terms of the chemical potential $\mu$ (or charge-carrier concentration) or the magnetic field intensity *B*, for fixed values of the remaining physical parameters. In the former description, it was demonstrated that the dc-magnetoconductivity response, which will be the focus of our attention, depends on the range of superlattice-potential and magnetic-field strengths considered^7–9^. In particular, it was found that the diagonal conductivity displays a strong anisotropy reversal when the magnetic field go from weak to intermediate strength, whereas the Hall conductivity exhibit plateaux for weak fields, which tend to disappear for intermediate ones. In the second description, most of the work focusses on the oscillations with *B* of the magnetoconductivity, especially on the Shubnikov-de Haas (SdH) and Weiss or commensurability oscillations^10–14^. It was shown theoretically that the Weiss oscillations in monolayer graphene are enhanced relative to those of ordinary 2D electron gas, and are more robust against temperature damping in the small magnetic field regime^10^. The experimental observation of commensurability oscillations in 1D-graphene superlattices (GSLs) was reported for the first time very recently^14^. The study of magnetoconductivity oscillations has been also carried out for other periodically modulated two-dimensional (2D) systems, such as bilayer graphene^15^ and phosphorene^16^.

Furthermore, if the periodic potential $V_{P}(x)$ in monolayer graphene is replaced by an aperiodic one *V*(*x*), such as Fibonacci, Thue-Morse and period-doubling potentials, it is expected that, due to the presence of exotic electronic spectra in the resulting 2D system, the corresponding magnetotransport properties may exhibit unusual characteristics. It is the purpose of this work to explore some of these characteristics, which, up to now, have not received a special attention. To carry out the study, we will use the Fibonacci GSL. This is because it represents, from a theoretical and experimental point of view, a simple and accessible quasiperiodic structure exhibiting fractal and self-similar properties not shared by other aperiodic systems. In particular, it was shown theoretically that the electronic spectra of GSLs exhibit a multifractal structure, which manifests in the properties of different transport quantities in the absence^17^ and presence^18^ of external fields. A further motivation to use Fibonacci GSLs is associated with the possibility of studying experimentally their magnetoconductivity characteristics, as occur for GaAs/AlGaAs Fibonacci lateral SLs^19^, where complicated commensurability oscillations of the magnetoresistence were observed.

The paper is organized as follows. In Sect. 2, we present the theoretical approach. Section 3 is devoted to the results and the corresponding discussions. Finally, the conclusions are presented in Sect. 4.

## Theory

As mentioned above, in this work we are concerned with the dc conductivity in quasiperiodic Fibonacci GSLs in a perpendicular magnetic field $\overrightarrow{B}=B{\widehat{z}}$. Specifically, we will focus our attention on the diffusive (band) $\sigma _{yy}$ and Hall $\sigma _{yx}$ conductivities, ignoring the collisional one, which is determined by transport through localized states. We use the Landau gauge $\overrightarrow{A}=(0,Bx,0)$ for the vector potential and take the Fibonacci SL potential *V*(*x*) along the *x*-axis.

To perform the calculations we will use the Kubo formula for $\sigma _{yy}$ and $\sigma _{yx}$, which, for non-interacting electrons in the LL representation, can be written down as^20,21^

1 $$\begin{aligned} \sigma _{yy}= & {} \frac{\beta \tau }{S}\sum \limits _{i}f(E_{i})[1-f(E_{i})]\left\langle i\left| J_{y}\right| i\right\rangle ^{2}, \end{aligned}$$

2 $$\begin{aligned} \sigma _{yx}= & {} -\frac{i\hslash }{S}\sum \limits _{i\text { }\ne \text { j}}\frac{ \left[ f\left( E_{i}\right) -f\left( E_{j}\right) \right] }{\left( E_{i}-E_{j}\right) ^{2}+\Gamma ^{2}}\left\langle i\left| J_{y}\right| j\right\rangle \left\langle j\left| J_{x}\right| i\right\rangle , \end{aligned}$$

where $S=L_{x}L_{y}$ is the SL area, $\beta =1/k_{B}T$, *f*(*E*) the Fermi distribution function, $J_{\alpha }=-ev_{F}\sigma _{\alpha }$ the $\alpha$-component of the current operator $\overrightarrow{J}$, $\sigma _{\alpha }$ the Pauli matrix, $\Gamma$ the LL broadening parameter, and $\tau$ the scattering time, which we assumed constant for all states. For low energy excitations around the *K* point of the Brillouin zone, $E_{\nu }$ and $\left| \nu \right)$, with $\nu =i,j$ in Eqs. (1) and (2), are the eigenvalues and eigenfunctions of the massless Dirac-like equation. We can then write $\left| \nu \right)$ in coordinate representation in the form^22^:

3 $$\begin{aligned} \left| \nu \right) ={\ }\left| n,k_{y}\right) = \frac{\exp (-iyk_{y})}{\sqrt{L_{y}}}\Phi _{nk_{y}}(x)\,, \end{aligned}$$

where $n=0,\pm 1,\pm 2,\ldots$ is the energy quantum number, $k_{y}$ is the conserved wavevector along the *y*-axis, and the spinor $\Phi _{nk_{y}}(x)$ satisfies the system of differential equations^22^:

4 $$\begin{aligned} {\widehat{h}}\Phi _{nk_{y}}(x)= & {} \left[ {\widehat{H}}_{0}+V(x)I\right] \Phi _{nk_{y}}(x)=E_{n}(k_{y}) \Phi _{nk_{y}}(x)\,, \end{aligned}$$

5 $$\begin{aligned} {\widehat{H}}_{0}= & {} \left( \begin{array}{cc} 0 &{} \hslash \omega _{c}{\widehat{a}}^{-} \\ \hslash \omega _{c}{\widehat{a}}^{\dagger } &{} 0 \end{array} \right) \,, \end{aligned}$$

*I* being the $2\times 2$ unit matrix, ${\widehat{a}}^{\pm }=(l_{B}/\sqrt{2})\left[ {\widehat{k}}_{x}\pm i(x-x_{0})/l_{B}^{2}\right]$ the raising and lowering operators, ${\widehat{k}}_{x}=-i\partial /\partial x$ and $x_{0}=k_{y}l_{B}^{2}$ the conserved center position.

It is clear that to calculate $\sigma _{yy}$ and $\sigma _{yx}$, for given values of $\Gamma$ and the chemical potential $\mu$, it is necessary to solve first the eigenvalue problem for the energy operator ${\widehat{h}}$, which is only possible if the Fibonacci SL potential *V*(*x*) is known. Here we suppose that *V*(*x*) is formed by rectangular barriers (layers *a*) and wells (layers *b*) arranged according to the Fibonacci sequence^23^:

6 $$\begin{aligned} W_{n}=\left\{ \begin{array}{l} W_{n-2}\,|\,W_{n-1}\,,\,\,\,\, \text {for}\,\,\,\,n\ge 3\,\,\text {and}\,\,\,\,\text {odd} \\ W_{n-1}\,|\,W_{n-2}\,,\,\,\,\, \text {for}\,\,\,\,n\ge 3\,\,\text {and}\,\,\,\,\text {even} \end{array} \right. , \end{aligned}$$

where $$W_{1}=a$$ and $$W_{2}=b$$. We assume that $$V(x)=V_{0}/2$$ in the barriers and $$-V_{0}/2$$ in the wells. Now, when using the potential *V*(*x*) associated with $$W_{n} $$ to solving Eq. (4), the origin of coordinates is taken at the inversion center of $${\overline{W}}_{n}$$, which is obtained from $$W_{n}$$ by removing its two extreme layers^24^.

It should be pointed out that the transformations $(ab\rightarrow a,abb\rightarrow b)$ and $(b\rightarrow a,ab\rightarrow b)$ transfer the element $W_{n}$ into $W_{n-2}$ and the reverse of $W_{n-1}$, respectively, indicating that the Fibonacci SLs exibit a fractal or self-similar structure. Consequently, to obtain self-similarity in the length scale^23^, it is necessary that $d_{b}/d_{a}=\tau =(1+\sqrt{5})/2$, where $\tau$ is the golden mean, $d_{a}$ the barrier width and $d_{b}$ the well width.

A simple inspection of Eq. (6) indicates that the spatial separation between adjacent interfaces is $d_a$ or $d_b=\tau \,d_a$, with $d_a$ and $d_b$ arranged according to a Fibonacci sequence. This means that $d_a$ ($d_b$) corresponds to a periodic spacing between interfaces with period $d_a$ ($d_b$). Since $d_a$ and $d_b$ are relatively irrational, a Fibonacci SL represents a quasiperiodic structure with two incommensurate periods.

## Results and discussion

At this point, it should be noted that, according to the above discussion, a quasiperiodic Fibonacci SL is an infinite self-similar structure generated according to $W_n$ for $n\rightarrow \infty$. In contrast to infinite periodic SLs, $W_\infty$ has not translational symmetry and an exact and systematic procedure to study its electronic properties does not exist. It is clear however that a good description of these properties can be achieved if $W_\infty$ is approximated by $W_n$ provided the order *n* is large enough. The latter requires that the spatial length $L(W_n)=d_a\,F_{n-2}+d_b\, F_{n-1}$ of $W_n$ must be sufficiently large in comparison with the corresponding magnetic length $l_B$, where the sequence $F_n=F_{n-2}+F_{n-1}$, with $F_1=F_2=1$, determines the Fibonacci numbers. It is easy to show, for instance, that the patterns $W_{15}$, $W_{17}$, and $W_{19}$, with the geometrical and physical parameters defined below, satisfies these conditions. Thus, any one of them can be used in the study of the corresponding electronic properties.

Numerical calculations are performed for the $W_{17}$ Fibonacci superlattice with $d_{b}/d_{a}=\tau$ and weak magnetic fields satisfying the inequality $l_{B}>>d$, where $d=d_{a}+\tau d_{b}$ is the average periodicity of the Fibonacci potential *V*(*x*). This allows us to use the quasi-classical approach to understand some of the results that will be presented below. We take $d=20\, \hbox {nm}$ and $V_{0}/E_{F}=2\pi$, where $E_{F}=\hslash v_{F}/d=33\, \hbox {meV}$ is an energy scale parameter^7^. With that value of *d*, it is easy to show that $d_{a}=d/(1+\tau ^{2})\approx 5.5\, \hbox {nm}$ and $d_{b}=d_{a}\tau \approx 9\, \hbox {nm}$.

**Figure 1:**
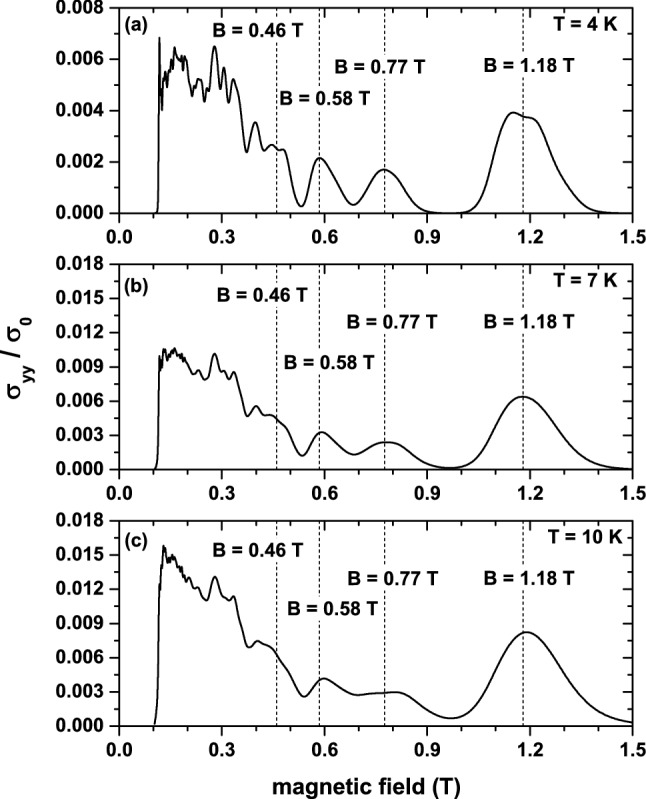
Diffusive conductivity (in units of $\sigma _0$) as a function of the applied magnetic field for several temperature values: (**a**) $T = 4$ K, (**b**) $T = 7$ K, and (**c**) $T = 10$ K, where $\sigma _0=(e^2/h)(4\beta \tau E_F^2/\hbar )$. Vertical dotted lines indicate the magnetic field intensities for which maxima have been obtained.

### Diffusive conductivity

Using the numerical solutions of Eqs. (4) and (3), we calculated the magnetic field dependence of the diffusive conductivity $\sigma _{yy}$ for different temperatures and chemical potentials $\mu$. Such a dependence is shown in Fig. 1 for weak magnetic fields, low temperatures and $\mu =0$. As can be seen, $\sigma _{yy}$ displays oscillations as a function of *B* whose characteristics depend on the range of magnetic fields considered. For the low-field range $(0.12\lesssim B\lesssim 0.36)$, $\sigma _{yy}$ exhibits a complicated oscillatory behavior, characterized by relatively rapid oscillations, whereas for the higher one ($0.36\lesssim B\lesssim 1.4$), these oscillations are much simpler and well-behaved.

Before presenting the analysis of these results, it is interesting to note that, as mentioned in the Introduction, similar complicated oscillations of the magnetoresistence were experimentally observed in GaAs/AlGaAs Fibonacci lateral SLs^19^. It was shown in that work, using a Fourier analysis, that these oscillations can be described by the superposition of a small number of incommensurate periodic (sinusoidal) modulations, with periods successively scaled by the golden mean $\tau$. The latter is a direct consequence of the self-similarity properties exhibited by Fibonacci SLs. Although this approach gives useful information about such oscillations, it does not relate them to the corresponding magnetic subbands, which can give further insight into the physical origin of these oscillations. Here we adopt the LL-energy approach to describe and understand the results depicted in Fig. 1.

**Figure 2:**
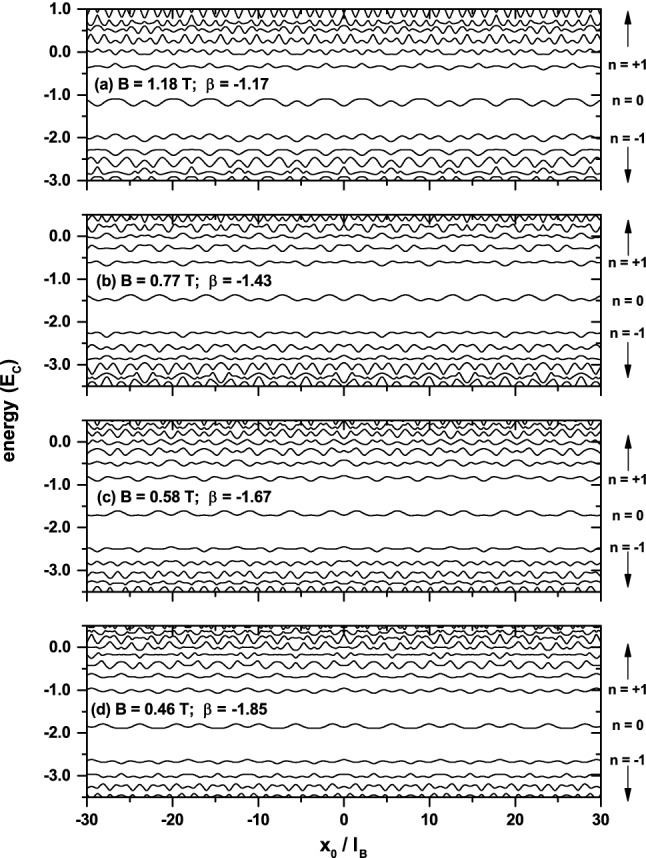
Center-position dependence and shifting $\beta (B)$ of the magnetic subbands corresponding to those magnetic fields at which the diffuse conductivity in Fig. 1 takes its maximum values. Note that the center of the $n = 2, 3, 4$, and 5 magnetic subbands for $B = 1.18$ T, 0.77 T, 0.58 T, and 0.46*T*, respectively, coincides with the $\mu =0$ chemical potential.

To carry out the study, it is necessary to take into account that^25^ the Fibonacci SL broadens the Landau levels of graphene into magnetic subbands and shifts all them rigidly downwards. The *B*-dependence of this shifting is given approximately by $\beta (B)=-v_{0}/\tau ^{3}$, with $v_{0}=V_{0}/\hslash \omega _{C}\approx 1/\sqrt{B}$ being the height of the barriers in units of the cyclotron energy. These properties are illustrated in Fig. 2 for $B=1.18$ T, 0.77 T, 0.58 T, and 0, 46 T, that are magnetic fields at which $\sigma _{yy}$ takes maximum values. Another important aspect is that, according to Eq. (1), if a magnetic subband is completely full or empty, its contribution to the diffusive conductivity vanishes exactly. This implies that, for sufficiently low temperature, the minimum values of $\sigma _{yy}$ occur at those magnetic fields for which the chemical potential $(\mu =0)$ is in between two magnetic subbands, whereas the maxima appear (approximately) when the center of the subbands crosses the chemical potential $(\mu =0)$, as shown in Fig. 2. The distribution of maxima and minima in the high-field range $(0.36\lesssim B\lesssim 1.4)$ are well defined because the corresponding magnetic subbands are energetically well separated (see Fig. 2). In contrast, the complicated oscillatory behavior observed in the low-field range is due to the overlap between the subbands.

**Figure 3:**
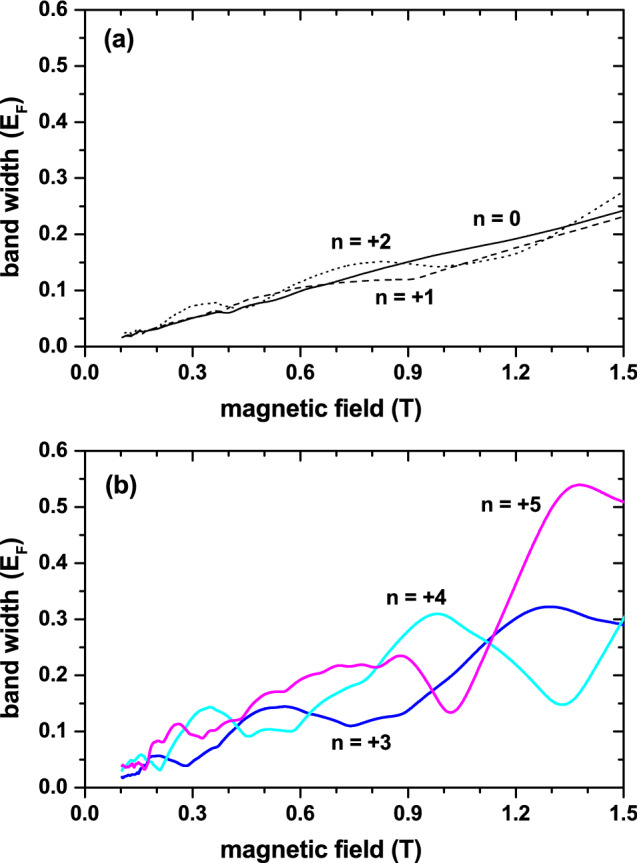
Magnetic field dependence of the band width for the $n = 0$, $n = +1$, and $n = +2$ magnetic subbandas (**a**). In (**b**), the same results but for $n =+3$, $n = +4$, and $n = +5$.

For a further characterization of the $\sigma _{yy}$ oscillations, the bandwidths of the $n\le 5$ magnetic subbands are depicted in Fig. 3. This choice is due to the fact that these are the subbands determining the oscillatory structure of $\sigma _{yy}$ in Fig. 1. Now, it is seen that the widths of the $n=0,1,2$ subbands increase with *B* and do not exhibit a clear oscillatory behavior, whereas those of the remaining ones increase non-monotonically with *B* exhibiting a slowly oscillating behavior. These bandwidth characteristics are in stark contrast to those of 1D periodic SLs, where the *B*-dependent width of the *n*-subband is proportional to the sum of two successive Laguerre polynomials^10,11,26^ and, therefore, exhibits oscillations with vanishing amplitude (minima) at certain values of the field. The number of such oscillations increases with *n*, especially in the range of weak magnetic fields, giving rise to the Weiss oscillations in the magnetoresistance, which originate from the commensurability between the SL period and the cyclotron diameter in the 1D periodic SL. This suggests that the oscillatory structure of $\sigma _{yy}$ shown in Fig. 1 cannot be associated with Weiss oscillations, but rather with Shubnikov-de Haas ones. This result is a direct consequence of the existence of two in-commensurate periods in Fibonacci SLs^27^.

Furthermore, since $l_{B}>>d$ we can carry out a quasiclassical analysis of the results presented in Fig. 1. In this approach, the band structure of the $W_{17}$ Fibonacci GSL in the absence of *B*, which can be calculated considering a periodic SL whose unit cell is based on the $W_{17}$ potential^28^, is not altered by the magnetic field, which only quantizes it and the charge-carrier motion is along equal energy curves in *k*-space. Under these conditions, we can assume that the cyclotron orbits in monolayer graphene are not substantially modified by the Fibonacci potential. Taking then into account that the band structure of graphene is given by $E(\overrightarrow{k})=\pm \hslash v_{F}k=\pm \hslash v_{F}\sqrt{ k_{x}^{2}+k_{y}^{2}}$, the area enclosed by the cyclotron orbit (circumference in $\overrightarrow{r}$-space) associated with the Landau level $E_{n}=sgn(n)\hslash \omega _{C}\sqrt{\left| n\right| }$ is given by

7 $$\begin{aligned} A_{n}=\pi R_{n}^{2}=\pi l_{B}^{4}\left( \frac{E_{n}}{\hslash v_{F}}\right) ^{2}=2\pi l_{B}^{2}\left| n\right| , \end{aligned}$$

where $R_{n}$ is the cyclotron radius.

As assumed above, Eq. (7) also applies to the case where the Fibonacci potential is considered, but replacing $E_{n}$ and the Fermi velocity $v_{F}$ by $E_{n}^{\prime }$ and $v_{F}^{\prime }$, respectively,

8 $$\begin{aligned} A_{n}^{\prime }=\pi R_{n}^{\prime 2}=\pi l_{B}^{4}\left( \frac{E_{n}^{\prime }}{\hslash v_{F}^{\prime }}\right) ^{2}=2\pi l_{B}^{2}\left( \frac{ E_{n}^{\prime }}{\hslash \omega _{C}}\right) ^{2}\left( \frac{v_{F}}{ v_{F}^{\prime }}\right) ^{2}, \end{aligned}$$

where $E_{n}^{\prime }/\hslash \omega _{C}$ represents the center of the shifted *n* magnetic subband corresponding to the LL $E_{n}/\hslash \omega _{C}=sgn(n)\sqrt{\left| n\right| }$ of monolayer graphene, with $n=0,\pm 1,\pm 2,\ldots$ being the Landau index. The velocity $v_{F}^{\prime }$ was introduced to include a possible small modification of the cyclotron orbits.

To compare the results obtained from Eqs. (7) and (8), it is necessary to take into account that $E_{n}/\hslash \omega _{C}$ is measured from the $n=0$ level. This implies that $E_{n}^{\prime }/\hslash \omega _{C}$ in Eq. (8) must be measured from the center of the shifted $n=0$ magnetic subband (see Fig. 2), i. e., $\varepsilon _{n}=E_{n}^{\prime }/\hslash \omega _{C}-\beta (B)$. Consequently, when the center of the *n* subbands crosses the $\mu =0$ chemical potential $\varepsilon _{n}=0$ and thefore $E_{n}^{\prime }/\hslash \omega _{C}=\beta (B)$. Considering now those magnetic fields *B* for which $\sigma _{yy}$ exhibits maxima and requiring that $A_{n}^{\prime }\approx A_{n}$, one obtains

9 $$\begin{aligned} \frac{v_{F}^{\prime }}{v_{F}}\approx \sqrt{\frac{\beta (B)^{2}}{\left| n\right| }}\,. \end{aligned}$$

The numerical evaluation of this ratio is straightforward if the shifting $\beta (B)=E_{n}^{\prime }/\hslash \omega _{C}=-(1.17,1.43,1.67,1.85)$ of the magnetic subbands, shown in Fig. 2, and the corresponding values $\left| n\right| =(2,3,4,5)$ of the Landau index are used in Eq. (9). This leads to the conclusion that such a ratio is independent of *n* and takes the value $v_{F}^{\prime }/v_{F}\approx 0.83\approx \tau /2$, indicating that one of the effects of the Fibonacci potential is to renormalize the Fermi velocity $v_{F}$.

Finally, one observes in Fig. 1 that when the temperature *T* increases, the peak structure of $\sigma _{yy}$ tends to broaden and to be suppressed, except the peak associated with the higher magnetic field ($B=1.18$ T), which is robust against temperature in the *T*-range considered. The latter behavior is due to the fact that the magnetic subband giving rise to that peak ($n = +2$ subband in Fig. 2a) is well separated from the nearest-neighbor ones, and the thermal excitations only slightly affect the corresponding peak structure, in contrast to the remaining peaks.

**Figure 4:**
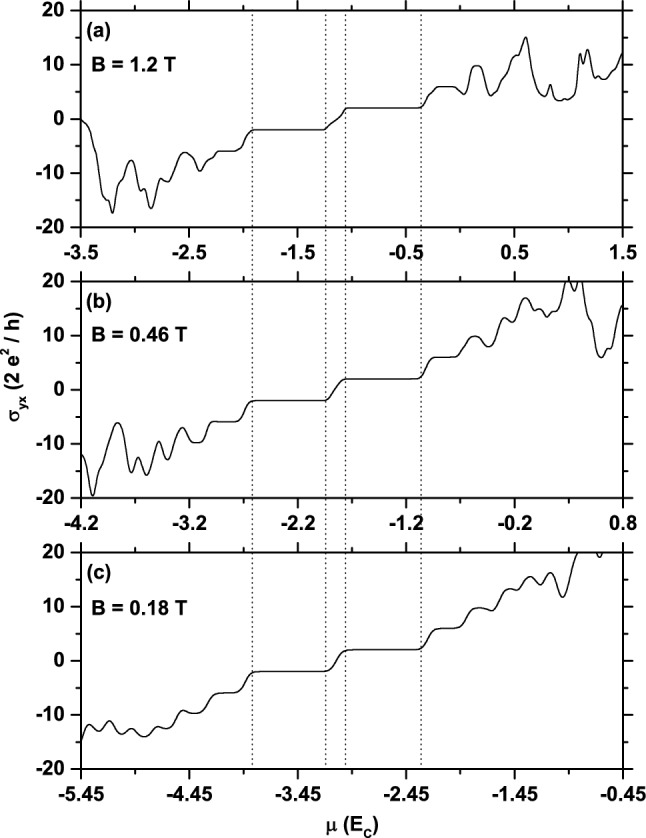
Hall dc-conductivity (in units of $2\,e^2/h$) as a function of the chemical potential (in units of $E_C=\hbar \omega _C$) for three values of the applied magnetic field, which are scaled by $\tau ^2$ and $\tau ^4$. The vertical lines determine the intervals for the plateaux that are around the $n=0$ shifted magnetic subbband.

### Hall conductivity

Let us now pay close attention to the dc Hall conductivity $\sigma _{yx}$, especially to its properties when the Fibonacci GSLs is subject to magnetic fields related by integer powers of the golden mean $\tau$. The latter choice guarantees similarity in the magnetic-field length scale of the structure^23^. That quantity computed from Eq. (2) is shown in Fig. 4 as a function of the chemical potential $\mu$ for $B=0.18$ T, $B^{\prime }=B\tau ^{2}=0.46$ T, $B^{\prime \prime }=B\tau ^{4}=1.2$ T, $V_{0}/E_{F}=2\pi$ and sufficiently low temperatures. We see that, for all these fields, $\sigma _{yx}$ exhibits well defined plateaux directly associated with the energy separation between the $n=0$, $\pm 1,\pm 2$ magnetic subbands. Notice that the center of steps when $\mu$ goes from holelike to electronlike carriers is situated at $\beta (B)=-1.14$, $-1.85$ and $-3.1$ in panels (a), (b) and (c), respectively, as required by the center positions of the corresponding $n=0$ magnetic sudbands. It is interesting to note that $\sigma _{yx}$ takes the unexpected value $\pm 4e^{2}/\hslash \left( 2N+1\right)$ on the visible plateaux, i. e., on the first $(N=0)$, second $(N=1)$ and third $(N=3)$ ones. This is an unexpected result because it does not follow the well-known sequence $\left( 4e^{2}/\hslash \right) N$, for conventional semiconductors, neither $\pm 4e^{2}/\hslash \left( N+1/2\right)$ for pristine graphene and periodic graphene SLs with only a Dirac cone in a weak field^9^. Thus, the half-integer quantum Hall effect does not occur in Fibonacci GSLs. It should be added that the sequence $\pm 4e^{2}/\hslash \left( 2N+1\right)$ was reported in Ref.^7^ to describe the Hall conductivity steps in periodic graphene SLs with additional Dirac cones. Since such cones do not exist in the energy spectra of Fibonacci graphene SLs, these arguments can not be invoked here to justify such Hall conductivity values at the mentioned plateaux. It is clear, however, that the occurrence of these plateaux are a direct consequence of the fractal properties of Fibonacci SLs, in particular, of the existence of two incommensurate periods in them^27,29^. Further works are needed for a better understanding of the Hall conductivity values at these plateaux.

Finally, a comparison between the results shown in Fig. 4a–c shows that the curves associated with the chosen fields exhibit a very similar behavior as functions of the scaled chemical potential $\mu /\hbar \,\omega _C$, where $\omega _C\sim \sqrt{B}$ is the cyclotron frequency. Indeed, it is apparent that if these figures are superimposed, such curves essentially coincide in that range of $\mu /\hbar \,\omega _C$ where the principal plateaux are located, whereas a slight difference is observed outside it. The latter is due to the overlap of the magnetic subbands. Thus, the Hall conductivity exhibits a self-similar structure for the considered magnetic fields, which are related by $\tau ^{2}$ and $\tau ^{4}$.

### Conductivity in periodic GSLs. A brief comparison

To compare our results with the corresponding ones for periodic GSL, it is necessary to take into account the properties of its energy spectra for the same parameters as those used above for the Fibonacci GSL, i. e., for barrier height $V_0/E_F=2\,\pi$ and magnetic field *B* such that $l_B\gg d$, where *d* is the SL period. It is well known that^9,30^, for these parameters, the magnetic subbands of the periodic SL are essentially flat, whereas its band structure in the absence of *B* only exhibits one Dirac point (cone).

Now, since $\langle i|J_y|i \rangle = \langle n,k_y|J_y|n,k_y \rangle \sim \partial E_n (k_y)/\partial k_y$ in Eq. (1) and the magnetic subbands are nearly flat, the contribution of the diffusive conductivity to the periodic-SL conductivity can be neglected, in contrast to the contribution shown in Fig. 1 for the Fibonacci GSL.

Further, due to the flat character of the magnetic subbands, the Hall conductivity exhibits well-defined plateaux around the mentioned Dirac point, with values given by $\pm (N+1/2)\,4\,e^2/h$ at these plateaux, as shown in Ref.^9^. These plateaux have the same origin as those shown in our Fig. 4, but the Hall conductivity values contrast to those of the Fibonacci GSL.

## Summary and conclusions

We have studied the magnetoconductivity properties of Fibonacci GSLs in the presence of a perpendicular magnetic field *B*. It was shown that the *B*-dependence of the diffusive conductivity exhibits a complicated oscillatory behavior whose charcateristics depend on the range of *B* considered and cannot be associated with Weiss oscillations, but rather with Shubnikov–de Haas ones. The absense of Weiss oscillations is attributed to the existence of two incommensurate periods in Fibonacci SLs. It was also found that the quasiperiodicity of the structure leads to a renormalization of the Fermi velocity $v_{F}$ of graphene. Our calculations revealed that, for weak *B*, the dc Hall conductivity $\sigma _{yx}$ exhibits well defined and robust plateaux as a function of the chemical potential, where it takes unexpected values. It was finally shown that $\sigma _{yx}$ displays self-similarity for magnetic fields related by $\tau ^{2}$ and $\tau ^{4}$.

## Data Availability

All the files with tables, figures, and codes are available. The corresponding author will provide all the files in case they are requested.
